# Optimal Experience in Adult Learning: Conception and Validation of the Flow in Education Scale (EduFlow-2)

**DOI:** 10.3389/fpsyg.2021.828027

**Published:** 2021-12-30

**Authors:** Jean Heutte, Fabien Fenouillet, Charles Martin-Krumm, Gary Gute, Annelies Raes, Deanne Gute, Rémi Bachelet, Mihaly Csikszentmihalyi

**Affiliations:** ^1^ULR 4354 - CIREL - Centre Interuniversitaire de Recherche en Education de Lille, Univ. Lille, Lille, France; ^2^Laboratoire Interdisciplinaire en Neurosciences, Physiologie et Psychologie, Université Paris Nanterre, Nanterre, France; ^3^Laboratoire VCR, Equipe d'accueil Religion, Culture et Société, École de Psychologues Praticiens de L'Institut Catholique de Paris, Paris, France; ^4^APEMAC UR 4360 Université de Lorraine, Metz, France; ^5^Institut de Recherche Biomédicale des Armées (IRBA), Brétigny, France; ^6^UNI-FlowLab, University of Northern Iowa, Cedar Falls, IA, United States; ^7^KU Leuven, Itec Research Group at Campus Kulak Kortrijk, Leuven, Belgium; ^8^Centrale Lille, University of Lille, Lille, France; ^9^Quality of Life Research Center, Claremont Graduate University, Claremont, CA, United States

**Keywords:** flow, optimal experience, autotelic experience, loss of self-consciousness, adult learning, adult education, motivation, well-being

## Abstract

While the formulation of Mihaly Csikszentmihalyi's theory of flow, including the experience dimensions, has remained stable since its introduction in 1975, its dedicated measurement tools, research methodologies, and fields of application, have evolved considerably. Among these, education stands out as one of the most active. In recent years, researchers have examined flow in the context of other theoretical constructs such as motivation. The resulting work in the field of education has led to the development of a new model for understanding flow experience in education, specifically dedicated to adult learning. As a result of both a meticulous analysis of existing models and consideration of more recent developments, a new flow scale has thus been developed. The aim of this study is therefore twofold: to validate the new flow measurement scale dedicated to the educational environment, EduFlow-2, and to test a new theoretical model. Students taking a course (*N* = 6,596), some on-site and others in a MOOC, participated. Several scales were administered online at the end of the participants' course during the 2017 academic year. The factor structure of EduFlow-2 was tested using Exploratory Structural Equation Modeling. Several models were tested. The model with a second-order factor best fit the data. We tested the invariance of the flow scale measure for gender and for the type of training (MOOC/on-site). We were able to show that the flow scale is invariant of the modalities of these two variables. Results revealed good psychometric qualities for the scale, making it suitable for both on-site and distance learning. The analysis also revealed significant relationships with the classic variables of motivation, self-efficacy, learning climate, and life satisfaction. Furthermore, all four dimensions of the model were found to be adequate and consistent with the underlying theoretical arguments. In the end, this new, short flow scale and the theoretical model were demonstrated to be promising for future studies in the field of education.

## Introduction

In January 1999, Ken Sheldon, Barbara Frederickson, Kevin Rathunde, and Mihaly Csikszentmihalyi came together for what was called the Akumal 1 Meeting in Akumal, Mexico, and produced the Positive Psychology Manifesto, which they revised during the Akumal 2 Meeting in January 2000. In the original version of the Manifesto, the authors laid the first foundations of this emerging field: The scientific study of optimal human functioning is to discover and to promote the factors that allow individuals and communities to thrive. In the revised version of the Manifesto, (Sheldon et al., 2000) stated that education is the first of the 6 core applications of positive psychology: “Improving child education by making greater use of intrinsic motivation, positive affect, and creativity within schools” (Sheldon et al., 2000, p. 1). However, we argue that this intention can be extended to lifelong learning, since the 4^th^ core application mentioned in the Manifesto regarding working life is “Improving work satisfaction across the lifespan by helping people to find authentic involvement, experience states of flow, and make genuine contributions in their work” (Sheldon et al., 2000, p. 1). It is therefore not surprising that the volume *Applications of Flow in Human Development and Education* has a particularly important place in *the Collected Works of Mihaly Csikszentmihalyi* (Csikszentmihalyi, 2014a,b,c).

### Mihaly Csikszentmihalyi, the Father of Flow Theory

Flow theory is one of the most significant theories of contemporary psychology. Csikszentmihalyi's pioneering work began with his examinations of creativity during his doctoral research as early as 1965 (Csikszentmihalyi, 1996, 2014a). He expanded his inquiry to the psychological determinants of subjective experience, then later to what constitutes a good life, finally focusing on flow (Csikszentmihalyi, 1975, 1990, 2014b). For more than a quarter of a century, his work grew in influence beyond a small community of researchers. Martin Seligman, during his term as president of the American Psychological Association (APA) (1998–2000), emphasized the importance of Csikszentmihalyi's work, notably by describing him as “the world leader in positive psychology research.” In the year 2000, Csikszentmihalyi received Brain Channel's Thinker of the Year Award.

Since 1990, his book *Flow: The Psychology of Optimal Experience*, published in 1990, has been translated into 23 languages. Csikszentmihalyi has probably become one of the most-cited psychologists in a variety of fields, including psychology, sports, education, arts, management, video games, online learning, and many others.

## Literature Review

With the aim of describing the growing interest in flow research in the new millennium, the European Flow-Researchers Network (EFRN) produced a scoping review (Peifer et al., 2021) following the steps proposed by Arksey and O'Malley (2005). The review included 258 quantitative and qualitative empirical studies published between 2000 and 2016. This review allowed us to correlate the themes identified in the review with the potential applications identified in the Positive Psychology Manifesto (Sheldon et al., 2000). The analysis classified “education” as the main topic, with 28.8% of the empirical studies published between 2000 and 2016 dedicated to this field of inquiry. Other topics representing at least 10% of published studies included arts/leisure (22.0%), health/psychotherapy/physiology (14.7 %), professional activities (11.6 %), physical activities/sport (11.0%). The works dedicated to education focused on pupils and education at the primary school level (8.2%), the secondary school/college level (20.4%), the continuing education level (69.4%), and teachers themselves (2.0%). Only 21.3% of education studies dealt with continuing vocational training/lifelong learning (vs. formal education, 78.7%).

Many researchers have also examined the link between flow experience and a wide range of motivational indicators: (a) engagement (e.g., Shernoff et al., 2003; Steele and Fullagar, 2009; Valenzuela and Codina, 2014; Mesurado et al., 2016), (b) goal orientation (e.g., Schüler et al., 2010; Oertig et al., 2014), (c) achievement motives (e.g., Engeser and Rheinberg, 2008; Busch et al., 2013), (d) interest (e.g., Bressler and Bodzin, 2013; Bachen et al., 2016), and (e) volition (e.g., Schattke, 2011). This is not surprising because many authors (e.g., Schüler et al., 2010; Schattke, 2011; Bassi and Delle Fave, 2012; Fulmer and Tulis, 2016) consider flow experience to represent a state of optimal motivation (Deci and Ryan, 2008; Heutte, 2019).

Flow in education has often been studied in combination with other theories. Many studies have examined the connection between flow and intrinsic motivation (Schüler et al., 2010; Keller et al., 2011; Valenzuela and Codina, 2014; Meyer et al., 2016). Flow is, in fact, often conceptualized as a theory of intrinsic motivation (Engeser and Rheinberg, 2008); however, studies have not yet reached consensus on whether or not intrinsic motivation is a necessary condition for flow in all contexts, or how the intrinsic motivation contributes to the dimensions of flow. The primary purpose of a meta-analysis of 28 studies (Fong et al., 2015) was to examine the relationship between Challenge-Skill Balance and flow, but the analysis also considered Challenge-Skill Balance and its possible relationship with intrinsic motivation. In the former, the correlation was moderate. In the latter, it was weaker still. Challenge-Skill Balance, combined with Clear Goals and Sense of Control, however, was a strong contributor to flow. Although the relationship between intrinsic motivation in the experience of flow and Deci and Ryan's (2008). Self-determination Theory, a prominent theory of intrinsic motivation, is not yet clearly established, Schattke (2011) did find that raising children in an environment that promotes self-determination helps them to engage in activities that will enhance flow experience.

Other studies on motivation and flow are linked to Social Cognitive Theory (SCT, Bandura, 2001). Results linking self-efficacy with flow frequency and higher levels of challenge and skill also show that self-efficacy predicts flow over time (Rodriguez-Sanchez et al., 2011; Heutte et al., 2016a). High levels of efficacy beliefs have a positive impact on flow experiences in academic settings (Salanova et al., 2006; Bassi et al., 2007; Heutte et al., 2016a). Various aspects of Bandura's (1986) self-regulation learning model were also shown to exert a significant and positive effect on flow state (Lee and LaRose, 2007; Rodriguez-Sanchez et al., 2011; Chen and Sun, 2016). Higher congruence between one's implicit motives and self-attributed motives is associated with better self-regulation, goal attainment and flow (Rheinberg and Engeser, 2012). Some studies highlight collective (or social) motivational conditions of flow and demonstrate that collective efficacy beliefs predict collective flow over time (Salanova et al., 2014).

To conclude this literature review, we can observe that various concepts raised by Deci and Ryan's SDT or Bandura's SCT seem to be excellent predictors of flow antecedents in educational contexts. Therefore, flow theory offers promising complementary perspectives for shedding more light on the psychological determinants of commitment and persistence in adult training and continuing education. It is for all these reasons that the perspectives concerning autotelic experience (well-being provided by the activity itself, rather than external rewards) in a lifelong learning context, although currently very little explored, seem particularly promising (Heutte, 2020).

### The Proposed Flow in Education Model

Engeser et al. (2021) argued that the definition of flow has changed very little since Csikszentmihalyi's (1975/2000) original formulation in 1975, and that there is strong agreement among researchers on the definition itself. Yet, they pointed out that there is a certain level of disagreement among researchers regarding how flow should be measured: “Indeed, over the past 35 years, researchers have kept developing and validating new measurement tools for flow, and modifying and re-validating established ones, which indicates that a gold measurement standard for flow has yet to be achieved” (Moneta, 2021; pp. 31–32).

This apparent paradox is not uncommon in the history of psychology and can be understood by recognizing that the path from the theoretical definition to the operationalization of a construct goes through the intermediate process of modeling. Thus, various tools have long been used to study flow in educational contexts (Table 1). However, to our knowledge, before the development of the first Flow in Education Model (Heutte et al., 2014a), there was no short multidimensional scale designed specifically for education.

**Table 1:** Some examples of tools used to study on flow in an educational context.

**Scales**	**Authors**	**NB items**	**NB dim**
Flow scale	Mayer (1978)	12	2
Flow questionnaire (Flow Q)	Csikszentmihalyi and Csikszentmihalyi (1992)	3	n.a.
Flow in human-computer interaction	Ghani and Deshpande (1994)	15	4
Flow state scale (FSS)	Jackson and Marsh (1996)	36	9
Flow in online environments	Novak et al. (2000)	66	13
Flow state scale-2 (FSS-2)	Jackson and Eklund (2002)	36	9
Dispositional flow scale-2 (DFS-2)	Jackson and Eklund (2002)	36	9
Flow-Kurzskala (FKS)	Rheinberg et al. (2003)	14	2
Work-related flow inventory (WOLF)	Bakker (2008)	13	3
EGameFlow	Fu et al. (2009)	42	8
Échelle de mesure du flow en éducation (EduFlow)	Heutte et al. (2014a)	12	4

*Adapted from Heutte (2019). n.a., not applicable*.

The Flow State Scale-2 (FSS-2) (Jackson and Eklund, 2002), measuring the nine conceptual dimensions of flow, is one of the most widely-used scales for studying optimal experience. However, Heutte et al. (2014b) were able to show that in an educational context, not all of the dimensions postulated by this scale were systematically captured by learners in self-report questionnaires. Thus, other studies, notably in French schools (Fenouillet et al., 2014) and in a MOOC (Massive Open Online Course), have made it possible to postulate a 4-dimensional structure (Heutte et al., 2016b).

The purpose of this study is, therefore, to propose the validation of a new scale dedicated to the educational environment for measuring flow. In accordance with the validation standards, several studies were carried out with a population of students at the INSPE (Institut National Supérieur du Professorat et de l'Education) of Lille (a Teacher Training Institute in the North of France) and in the Project Management MOOC (MOOC GdP) developed by a team of volunteers, startups and Centrale Lille (an engineering Grande École in North of France) in order to obtain an experimental version. Then, a series of analyses were carried out to explore its factorial structure and to confirm it. The construct validation was completed in a study of concurrent validity and correlates.

## Methods

### Participants

The sample (N = 6,596) had an age of 30.01 (Max = 69, Min = 18, SD = 9.41). Men (*n* = 2,947) and women (*n* = 3,225) (gender data missing for 397) were students at the INSPE of Lille (*n* = 3,232) and students enrolled in a MOOC (*n* = 4,264).

### Measures

Multiple measures were used in the course of this study:

- EduFlow-2 (12 items) with four subscales including Cognitive control (FlowD1, three items), Immersion and Time transformation (FlowD2, three items), Loss of self-consciousness (FlowD3, three items) and Autotelic experience—well-being provided by the activity (FlowD4, three items).
- The Generalized self-efficacy scale (10 items) in academic activities (adapted from Schwarzer and Jerusalem, 1995; French of GSES, Heutte, 2011).
- Learning Climate Questionnaire [6-item French version of LCQ, Leroy et al., 2013; adapted from Williams and Deci (1996)].
- Satisfaction with Life Scale (5-item French version of SWLS, adapted from Diener et al., 1985; Blais et al., 1989).
- French *Adult Education Motivation Scale* (FAEMS, Fenouillet et al., 2015), with 24 items in six subscales, including Intrinsic Motivation to know (IMk, four items), four types of extrinsic motivation [i.e., integrated (MEinteg, four items), identified (MEident, four items), introjected (MEinteg, four items) and external (MEext, four items), and amotivation (AM, four items)].

### Procedures

During the phase of the study conducted at the INSPE of Lille, students were given a survey asking them to evaluate the quality of training, teaching, and administration [in French “évaluation de la qualité des formations, des enseignements et du fonctionnement” (EQFEF)] (Heutte and Ghouch, 2018). The procedure was described in a specific document: the EQFEF charter available on the Institute's website. At the time of administrative registration, in September 2016, information regarding the gender and age of all students was collected. At the end of the second semester of the academic year, in June 2017, the GSES, LCQ, SWLS and EduFlow-2 scales were completed. Consent of the participants was obtained at the beginning of the survey, after informing all students that they should read the EQFEF charter and that participation in the study was voluntary.

One week before the start of the MOOC in September 2016, information about the gender, age and status of the participants (full-time student or working person) was collected. At the end of the fourth week of the MOOC, the FAEMS and EduFlow-2 scales were completed.

All questionnaires were administered online on a server hosted and secured by the University of Lille (LimeSurvey Version 2.50+ Build 160620).

## Results

Descriptive statistics were performed with R 4.0.4 (package Psych 2.09) and Jamovi 1.2.27. Confirmatory analyses and structural equation modeling were performed with Mplus version 8.5.

### Factorial Analysis

To validate the factor structure of the EduFlow-2 scale, we used Exploratory Structural Equation Modeling (ESEM) as advocated by various authors (Asparouhov and Muthén, 2009; Marsh et al., 2013, 2014). To clearly establish the factor structure, while it is acceptable for items to be weakly correlated with all factors (λ <0.4), it is essential that the correlation is stronger with the factor that represents the underlying psychological construct.

Model fit was tested with the following indicators and their recommended cut-off values (Browne and Cudeck, 1992; Kahn, 2006; Worthington and Whittaker, 2006; Hooper et al., 2008): a comparative fit index (CFI) and the Tucker Lewis index (TLI) above.90; a Root Mean Squared Error of Approximation (RMSEA) below.08, with an upper confidence interval which should not exceed.08 (Hu and Bentler, 1999); a Standardized Root Mean Squared Residual (SRMR) below.08; a chi-square to degree of freedom ratio preferably under 3.

Indicators fit are all above the expected threshold even if the χ2 remains significant (χ2(24) = 227.072, *p* < 0.001; CFI = 0.99; TLI = 0.99; RMSEA = 0.036 [CI 90% 0.032 0.041]; SRMR = 0.007). Furthermore, the results of the correlations between factors and items are broadly in line with our expectations. No item correlated with more than one factor above 0.40 (Table 2). The three D1 items all have correlations above 0.40 on the first factor. It can, therefore, be stated that factor D1 represents cognitive control. The three D2 items have correlations above 0.40 only on the second factor, so this factor represents immersion. The three D3 items all correlate above 0.80 on factor 3, so it can be stated that this factor represents lack of concern about the self. Finally, the D4 items all have correlations above 0.70 on the fourth factor, so it can be stated that this factor represents autotelic experience (well-being provided by the activity itself). Finally, the internal consistency of each dimension is good since the omega coefficient is >0.70 for each of the four dimensions (Table 2).

**Table 2:** Results of exploratory structural equation modeling, omega and descriptive statistics.

	**1**	**2**	**3**	**4**	**N**	**Mean**	**SD**	**Skewness**	**Kurtosis**
D1a	0.67	0.05	−0.02	−0.04	6,568	5.03	1.27	−0.50	0.40
D1b	0.73	−0.04	0.04	0.05	6,567	4.85	1.36	−0.47	0.15
D1c	0.60	0.07	0.03	0.04	6,487	4.89	1.41	−0.46	−0.10
D2a	0.03	0.82	0.01	−0.03	6,579	5.02	1.47	−0.61	0.01
D2b	0.16	0.72	−0.02	0.05	6,527	5.14	1.40	−0.64	0.15
D2c	−0.13	0.45	0.02	0.37	6,564	4.73	1.78	−0.48	−0.65
D3a	0.04	0.03	0.78	−0.03	6,589	5.14	1.86	−0.76	−0.48
D3b	−0.02	0.00	0.92	0.01	6,584	5.14	1.83	−0.74	−0.50
D3c	0.00	−0.01	0.89	0.02	6,495	5.22	1.78	−0.79	−0.39
D4a	0.08	0.02	0.01	0.81	6,564	4.37	1.68	−0.26	−0.63
D4b	0.06	−0.04	0.00	0.89	6,554	4.29	1.66	−0.22	−0.65
D4c	−0.04	0.08	−0.02	0.73	6,434	4.27	1.74	−0.22	−0.78
ω	0.74	0.80	0.90	0.89					

Table 3 allows us to evaluate the relationships between the different dimensions of flow measured by our scale and other psychological dimensions measured by other scales. Overall, the correlations are in the expected direction. The SEGS (Self-efficacy in academic activities) LCQ (Learning Climate) and LSWS (Satisfaction with Life) scales all have positive and significant correlations with the four dimensions of the EduFlow-2 scale (Table 3). It also appears that the different dimensions of the French Adult Education Motivation Scale (FAEMS) do not show the same correlations with the EduFlow-2 dimensions. As expected, amotivation is negatively correlated with all EduFlow-2 dimensions. The two forms of controlled motivation, external regulation and introjected regulation, are also more weakly-correlated than are the three forms of self-determined motivation (identified regulation, integrated regulation and intrinsic motivation).

**Table 3:** Correlations between flow dimensions and measurement scales and descriptive statistics.

	**N**	**Flow D1**	**Flow D2**	**Flow D3**	**Flow D4**	**Mean**	**SD**	**ω**
Flow D1	6,436	—				4.93	1.09	0.74
Flow D2	6,339	0.39***	—			4.96	1.31	0.80
Flow D3	6,349	0.33***	0.15***	—		5.17	1.66	0.90
Flow D4	6,267	0.41***	0.65***	0.12***	—	4.32	1.51	0.89
SEGS	2,268	0.66***	0.33***	0.43***	0.43***	4.93	1.03	0.94
LCQ	2,164	0.43***	0.40***	0.16***	0.50***	4.72	1.37	0.94
SWLS	762	0.41***	0.27***	0.31***	0.33***	4.95	1.24	0.87
AM	4,132	−0.23***	−0.44***	−0.08***	−0.39***	1.41	0.70	0.84
MEext	4,142	0.08***	0.09***	−0.08***	0.20***	2.81	1.08	0.83
MEintr	4,131	0.02***	0.26***	−0.06***	0.37***	2.76	1.13	0.88
MEident	4,137	0.22***	0.41***	0.03***	0.48***	3.91	0.88	0.84
MEinteg	4,140	0.20***	0.43***	−0.06***	0.63***	2.86	1.06	0.92
MIk	4,139	0.26***	0.53***	0.09***	0.60***	3.93	0.87	0.86
SDI	4,130	0.29***	0.57***	0.10***	0.60***	8.84	5.45	

*** p < 0.001. FlowD1, cognitive control; FlowD2, immersion and time transformation; FlowD3, loss of self-consciousness; FlowD4, autotelic experience; SEGS, self-efficacy in academic activities; LCQ, learning climate; SWLS, satisfaction with life scale; AM, a motivation; MEext, external regulation; MEintr, introjection; MEident, identification; MEinteg, integration; MIk, intrinsic motivation to know; SDI, self-determination index.

We also note that, although very weak, there are negative correlations between the EduFlow-2 dimension D3, Loss of self-consciousness, and identified regulation (*r* = −0.06, *p* < 0.001) and integrated regulation (*r* = −0.09, *p* < 0.001). These correlations, which run counter to our expectations, can be explained by the learning context in which these measures were taken, which does not favor social relations and therefore this dimension of flow.

**Figure 1:**
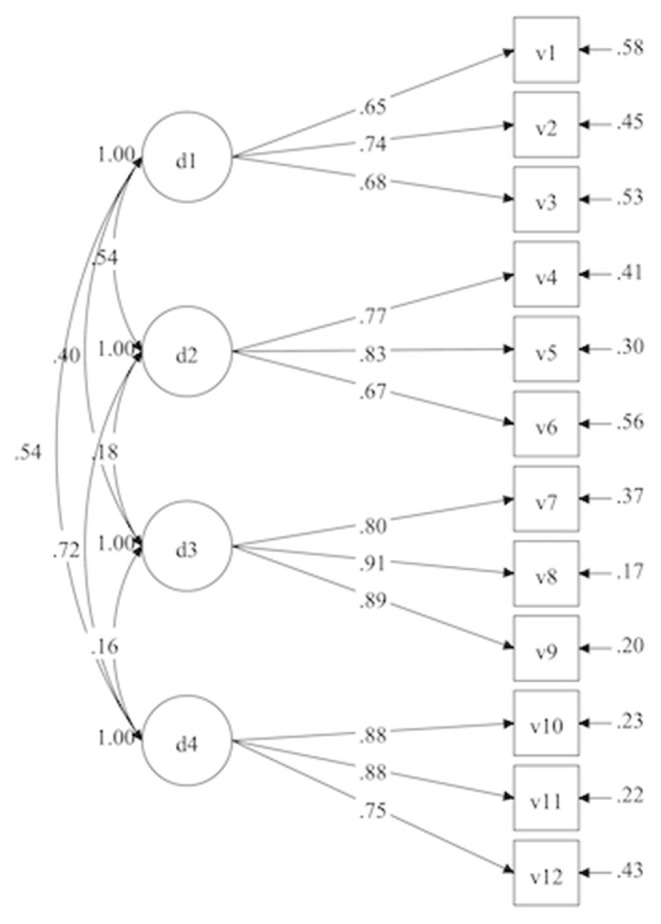
Measurement model.

We tested different relationships between the four dimensions of our scale that we identified earlier. The fit indicators of these models are compared to the fit of the measurement model (Figure 1) which is presented in Table 4.

**Table 4:** Fit indicators for the assessed models.

**Model**	**χ^2^**	**df**	**RMSEA**	**SRMR**	**CFI**	**TLI**	**AIC**	**BIC**
Measure. model	1,052.518	48	0.057	0.031	0.974	0.964	248,676.438	248,960.442
Model cond. State	2,476.884	51	0.086	0.078	0.937	0.919	250,094.763	250,358.482
Sec order factor	1,494.729	50	0.067	0.057	0.963	0.951	249,114.648	249,385.129

*Measurement Invariance for sample and gender*.

With regard to the criteria stated above, the three models presented a correct fit to the data. However, if we compare the models with each other, it appears that the models do not all have the same fit indicators.

**Figure 2:**
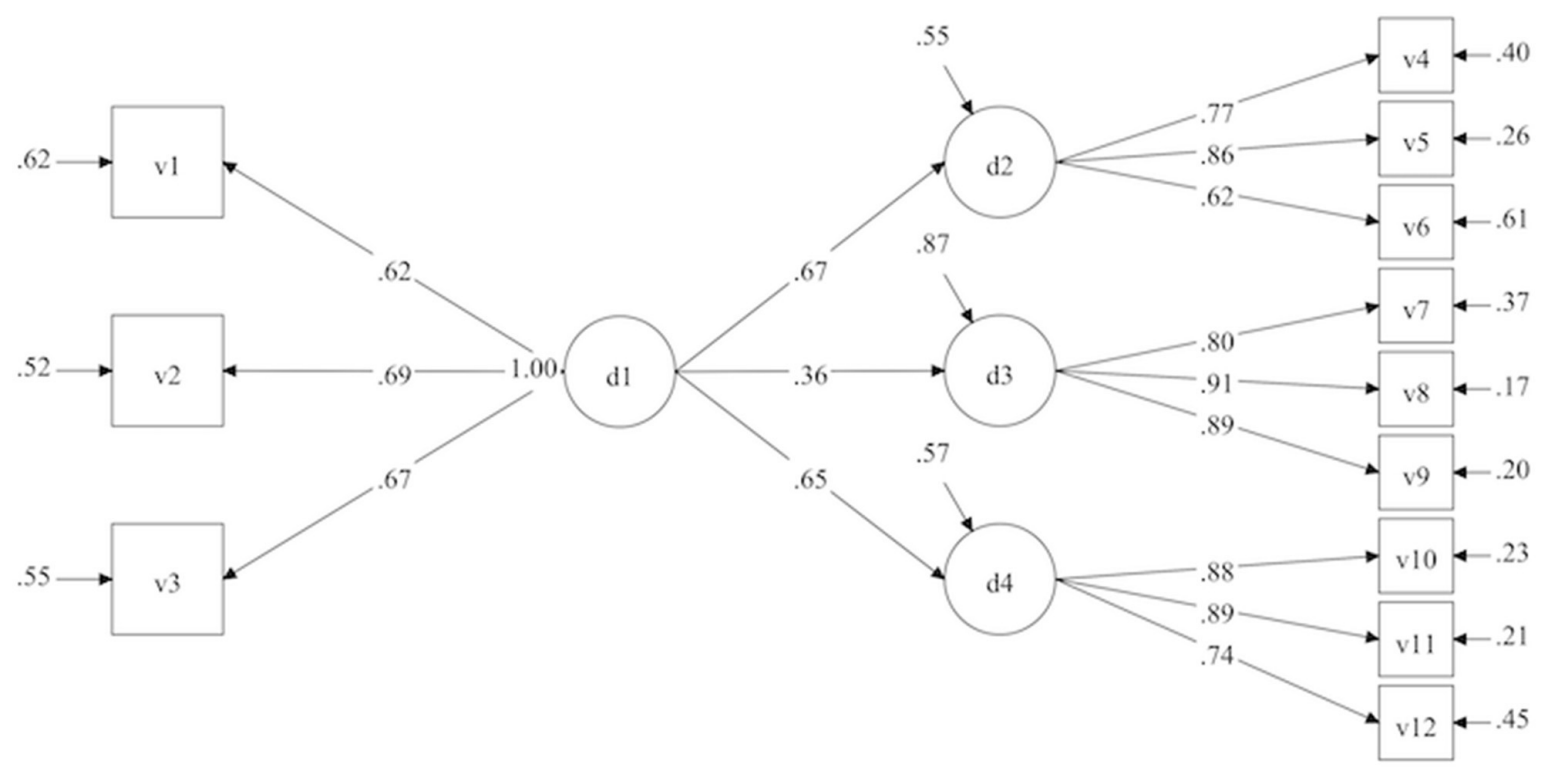
Model of flow condition and state.

The flow condition and state model (Figure 2) is based on Kawabata and Mallett's (2011) model, which distinguishes flow conditions from flow state. From this perspective this model states that Flow D1 (Cognitive Control), which is considered a necessary precondition for flow in education, will have an effect on FlowD2 (Immersion and Time Transformation), FlowD3 (Loss of self-consciousness), and FlowD4 (Autotelic Experience), which together create the state of flow. As we can see (Table 4), this model fits the data but it is the one that deviates most from the measurement model.

**Figure 3:**
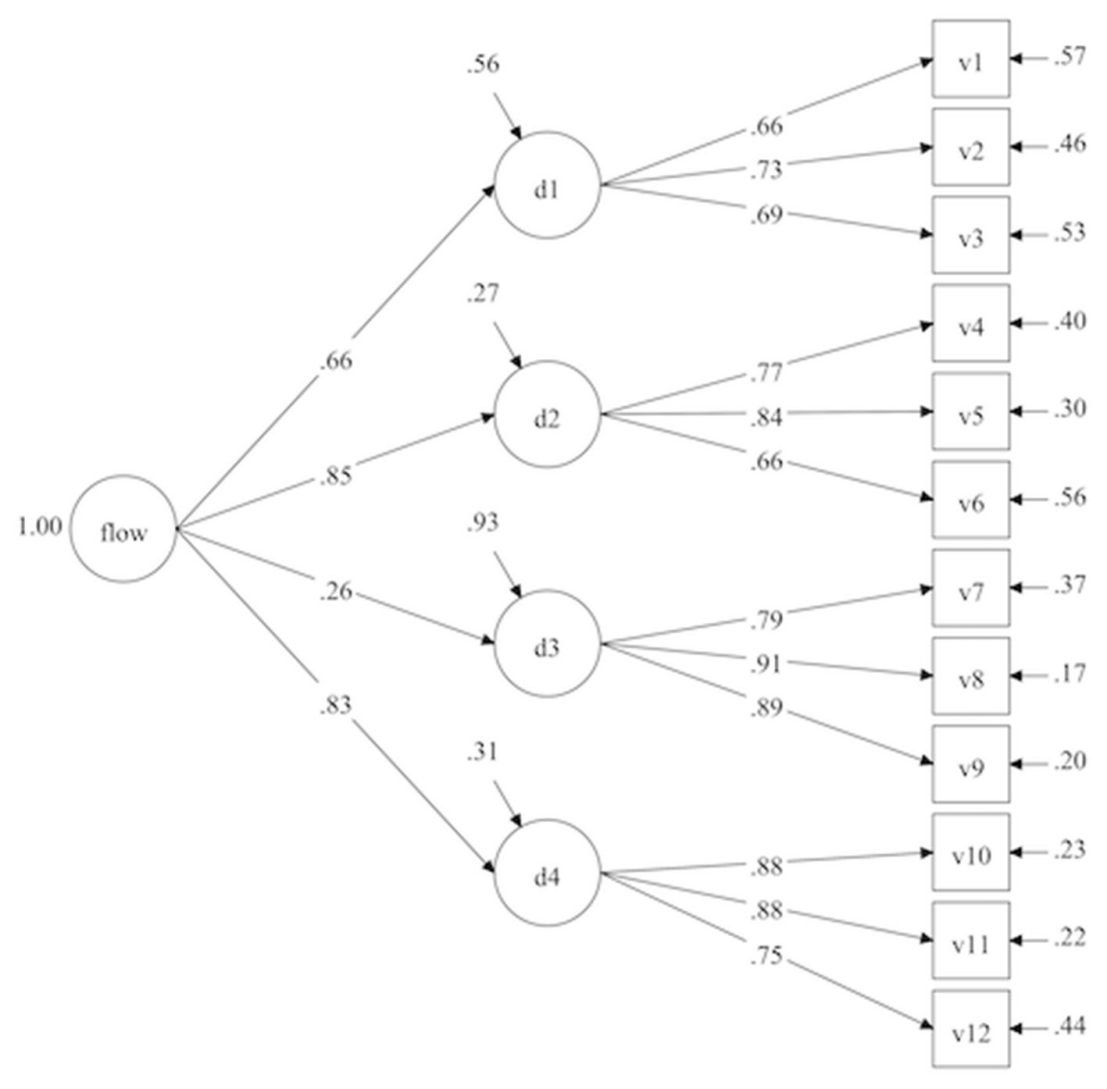
Second order factor.

The second order model (Figure 3) postulates the existence of a second order factor which would correspond to a general flow factor in education. Again this model fits the data correctly. However, the measurement model fits better than the other two. We can also see that the model with a second order factor is a better fit than the flow condition and state model (Table 4).

In order to assess whether responses of the scale differ by sample and differ by gender, we first tested the measurement invariance for these variables (Van de Schoot et al., 2012). In order to test the measurement invariance, we first tested the configurational invariance that allows us to establish the baseline measure for all groups. We then tested for metric invariance by constraining the coefficients of the latent factors to be equivalent across groups. We then proceeded to constrain the intercepts in addition to the coefficients of the latent factors, which corresponds to scalar invariance.

To test invariance, it was necessary to compare the different forms of invariance (configurational, metric, scalar) with each other. However, comparison methods based on the χ^2^ are sensitive to the sample size (Chen, 2007), so we relied on the difference between the fit indicators. Cheung and Rensvold (2002) recommended using a ΔCFI value >0.01 as a threshold for asserting a significant drop in fit between models. Chen (2007) also recommended using the ΔCFI, ΔRMSEA and ΔSRMR. To take these different recommendations into account, we considered the following thresholds for estimating measurement invariance, ΔCFI ≤ 0.01, ΔRMSEA ≤ 0.015 and ΔSRMR ≤ 0.03 for metric invariance and ΔCFI ≤ 0.01, ΔRMSEA ≤ 0.015 and ΔSRMR ≤ 0.01 for scalar invariance.

Table 5 summarizes the set of fit indicators for the configural, metric and scalar invariance for measurement invariance for the educational flow scale with respect to the sample (student/MOOC). As we can see, all the comparison indicators allow us to say that there is measurement invariance between flow in the MOOC and in the classroom. The same observation can be made when comparing men and women. The flow is invariant between men and women (Table 6).

**Table 5:** Fit indicators to assess the invariance of the EduFlow-2 scale measure for the sample.

**Model**	**χ^2^**	**Df**	**CFI**	**TLI**	**RMSEA**	**SRMR**	**ΔCFI**	**ΔTLI**	**ΔRMSEA**	**ΔSRMR**
Configural	1,178.828	96	0.97	0.96	0.059	0.033				
Metric	1,237.211	104	0.97	0.96	0.058	0.037	0.001	0.002	0.001	0.004
Scalar	1,556.28	112	0.96	0.96	0.064	0.041	0.008	0.007	0.006	0.004

**Table 6:** Fit indicators to assess the invariance of gender on the EduFlow-2 measure.

**Model**	**χ^2^**	**Df**	**CFI**	**TLI**	**RMSEA**	**SRMR**	**ΔCFI**	**ΔTLI**	**ΔRMSEA**	**ΔSRMR**
Configural	1,094.298	96	0.97	0.96	0.058	0.032				
Metric	1,120.092	104	0.97	0.97	0.056	0.034	0.001	0.002	0.002	0.002
Scalar	1,179.499	112	0.97	0.97	0.055	0.034	0.001	−0.001	−0.001	0

## Discussion

### The Strength of the EduFlow-2 Model

At their core, the results reveal satisfactory psychometric qualities. Our results show that it was possible to identify a model that fist the data with an internal consistency in line with psychometric standards. Furthermore, the examination of the invariance of the measure confirmed that the scale could be used both in the context of a MOOC and in the classroom, as well as and regardless of gender. These different results attest to good psychometric qualities that will allow the scale to be used in future studies, both in classrooms and in MOOCs, and for both women and men. In other words, the model (Figure 2, Tables 2, 4–6) underpins the EduFlow-2 Scale construct. The EduFlow-2 Scale has three main advantages:

- It suits flow measurement in various educational contexts;
- It is a short tool, reducing respondent burden;
- It highlights the difference between four dimensions of flow that are strongly and significantly related to many psychological determinants of motivation and volition in learning contexts, such as self-efficacy in academic activities; Learning Climate; Satisfaction with Life scale; self-determination, including Intrinsic Motivation (Table 3).

Beyond validating the scale itself, the results focused our attention on the role played by Loss of self-consciousness (D3) in the development of the self and in the development of relationships with others, an intriguing relationship discussed in the following section.

### Loss of Self-consciousness

While Loss of self-consciousness (D3) was strongly correlated with self-efficacy in Academic Activities and with Satisfaction with Life, and was negatively correlated with Amotivation, correlations with several motivation scales were weak, a finding that is not surprising given the mixed literature on both the influence of intrinsic motivation during flow and on the role that context plays in contributing to motivation during flow. Since Loss of self-consciousness has not been the focus of extensive empirical study, there remain under-explored issues relevant both to the EduFlow-2 and to flow research more generally.

### The Issue of Construct Definition

A close reading of Csikszentmihalyi's theoretical discussions (Csikszentmihalyi, 1975, 1990, 1993), reveals his vision of not a single factor, but of a multidimensional and dynamic variable, potentially one contributing to the development of the Self and even to one's relationships with others. He enumerates several ways (Csikszentmihalyi, 1975), from the concrete and personal to the abstract and social, that the variable can be described: loss of ego, self-forgetfulness, transcendence of individuality, and fusion with the world (p. 42). Existing scales that measure individual flow (e.g., Jackson and Eklund, 2002; Heutte et al., 2016b) operationalize the variable as one that is concrete and personal. Psychic energy is so consumed by a challenging activity that no bits of attention remain for considering how an outside Other might be evaluating you.

Fidelity in measuring Loss of self-consciousness will improve following research that better delineates the dimension from Merging of Action and Awareness. While Loss of self-consciousness can happen without the outside Other, Csikszentmihalyi's descriptions sometimes blur the two dimensions, describing Loss of self-consciousness in Japanese motorcycle gang members who report feeling as if they are “one flesh” with other cyclists during a run (p. 63); surgeons who describe becoming “a single organism, moved by the same purpose” (p. 65), in a networked connection with others in the operating room; and climbers who develop a sense of kinship “between fingers and rock, between the frail body and the context of stone, sky, and wind” (Csikszentmihalyi, 1990, p. 64). Becoming part of a system greater than oneself elevates Loss of self-consciousness well beyond the concrete and personal.

### The Issue of Construct Power

Csikszentmihalyi's abstract and social view of Loss of self-consciousness—as transcendence of individuality and fusion with the world—advances the power of the construct radically beyond not feeling judged by the Other to feeling integrated with all Others. The perspective bears thematic similarity to Erikson's (1980) argument that adulthood brings the critical developmental crisis of developing Generativity (“establishing and guiding the next generation”) (p. 103) vs. Stagnation. Csikszentmihalyi's bold claim is that the Loss of self-consciousness grows more sophisticated as one's complexity increases. Flow theory holds that complexity increases incrementally each time a person experiences flow.

### The Issue of Context

Flow measurement has established (Procci et al., 2012) that context and variations in activities can play a significant role in correlations between factors. Heutte et al. (2014b) noted that several dimensions are not experienced at measurable levels by learners in formal educational settings. For example, because students in a MOOC work with limited interaction, and often independently, there is little opportunity for a Loss of self-consciousness since there is no Other to judge them while doing their work. In contrast, high school students actively engaged in experiential learning environments do report a Loss of self-consciousness (Gute and Csikszentmihalyi, 2018).

Csikszentmihalyi's bold and optimistic theoretical foundation for Loss of self-consciousness has set the stage for necessary and significant empirical research that will greatly aid the measurement of flow in learning, particularly of adult learners. As a next step, we recommend that Loss of self-consciousness should become a focus in additional research, in order to build on this study by investigating the experience in a range of learning contexts.

### Additional Research Avenues Incorporating Learning and Classroom Analytics

EduFlow-2 is a short multidimensional scale specifically designed for education and for allowing repeated measures over time. Self-reporting measures offer several fundamental strengths in measuring the latent variable of interest: e.g. high construct validity, ease of interpretation, inexpensive and relatively quick administration (Kline, 1993). For these reasons, self-reports are often used as the gold standard for the latent variable under investigation, flow in this context. Yet, next to these strengths, self-reports are also prone to some fundamental limitations. Self-reporting requires participants to remember and assess their own emotional/motivational state, which depends on the person's emotional intelligence. Pirsoul et al. (2019) claimed that participants cannot capture their unconscious emotions. Self-reporting scores may also be biased because of the way in which the question is specified, or the way in which the answering options are formulated. Although Pekrun (2020) argued that self-report is indispensable for a nuanced assessment of mental states, he stressed that self-reports can be influenced by social desirability and can also be influenced by culture, an effect that is mainly attributed to differences in semantic understanding.

Next to the aforementioned fundamental limitations, self-reports are also prone to certain more practical limitations. For instance, the traditional approach of using self-reports to directly measure latent variables has a limited temporal resolution, as it is not possible to survey participants on a permanent basis. It is also not possible to automate self-reports, as the involvements of participants in self-reports is obviously necessary. Furthermore, when self-reports are collected during learning, participants' activities need to be interrupted, which can affect natural behavior and learning.

The combination of these practical limitations, together with the emergence of new monitoring technologies, has motivated innovative research projects to explore the possibility of directly measuring manifest variables that could serve as proxies for the latent variable of interest.

Several new technologies enable capture of new types of data: behavior within online learning environments or MOOCs can be tracked by logging clickstream data (e.g., Coussement et al., 2020), images can be analyzed by applying computer vision techniques on video-recordings (e.g., Raca et al., 2014; Vanneste et al., 2021), psycho-physiological data can be measured via wearable devices (e.g., Vanneste et al., 2020), and eye-measures can be monitored by eye-tracking and pupillometry (Van Acker et al., 2020).

These new tools not only provide opportunities to measure emotional/affective engagement (including flow), in real-time, they also creates opportunities to investigate optimal learning environments (Shernoff and Csikszentmihalyi, 2009). While MOOCs are remarkable environments for the development and validation of measurement tools from a methodological standpoint, they are not well-suited for the study of the social dimensions of learning. In these massive multi-learner online environments, learners are very often massively alone. MOOCs are therefore not the most suitable environments to study the optimal learning environment from the social-conative perspective (Heutte, 2017, 2019, 2021). In future research, we hope to investigate flow as one of the important factors within authentic learning settings—ecosystems in which learning happens in interaction with the content, the tools, the peers, the space, and the teacher. We aim to go beyond current work by defining and assessing quality of teaching and learning in terms of measurable multimodal indicators based on behavior, log data, eye tracking data, audio-visual data and sensor data from activities within the learning spaces. This means that we will not only focus on learning analytics, but also on modeling the skills developed by the learners using educational software. It has been stressed in the current educational literature (e.g., Prieto et al., 2016; Martinez-Maldonado et al., 2021) that we should broaden the scope of analytics, modeling not only the learners' interactions with digital tools, but also anything that may happen in this specific ecosystem called a classroom or learning space (Dillenbourg, 2021).

## Conclusion

Our results revealed good psychometric qualities of the EduFlow-2 scale make it suitable for both on-site and distance learning. They also revealed significant relationships with the classic variables of motivation, self-efficacy, learning climate, and life satisfaction. Furthermore, all four dimensions of the model were found to be adequate and consistent with the underlying theoretical arguments. In the end, this new short, multidimensional flow scale and the theoretical model behind it will thus be very useful for the study of optimal learning experience in many contexts, in particular for longitudinal researchers wishing to study the interaction effects between flow and other theoretical concepts that may support lifelong learning.

## Data Availability Statement

The raw data supporting the conclusions of this article will be made available by the authors, without undue reservation.

## Ethics Statement

Ethical review and approval was not required for the study on human participants in accordance with the local legislation and institutional requirements. The patients/participants provided their written informed consent to participate in this study.

## Author Contributions

JH, FF, CM-K, MC, and RB contributed to the conception and design of the study. RB organized the database. FF carried out the statistical analysis. JH, FF, CM-K, and MC wrote the first draft of the manuscript. GG, AR, RB, and DG wrote sections of the manuscript. All authors contributed to the revision of the manuscript, read, and approved the submitted version.

## Dedication

This research is dedicated to the memory of Mihaly Csikszentmihalyi, who provided inspiration and collaboration in the development of this study.

## Funding

This project was supported by the French Government through the Programme Investissement d'Avenir (I-SITE ULNE / ANR-16-IDEX-0004 ULNE) managed by the Agence Nationale de la Recherche.

## Conflict of Interest

The authors declare that the research was conducted in the absence of any commercial or financial relationships that could be construed as a potential conflict of interest.

## Publisher's Note

All claims expressed in this article are solely those of the authors and do not necessarily represent those of their affiliated organizations, or those of the publisher, the editors and the reviewers. Any product that may be evaluated in this article, or claim that may be made by its manufacturer, is not guaranteed or endorsed by the publisher.
