# Corrigendum: Optimal experience in adult learning: Conception and validation of the flow in education scale (EduFlow-2)

**DOI:** 10.3389/fpsyg.2022.894527

**Published:** 2022-12-07

**Authors:** Jean Heutte, Fabien Fenouillet, Charles Martin-Krumm, Gary Gute, Annelies Raes, Deanne Gute, Rémi Bachelet, Mihaly Csikszentmihalyi

**Affiliations:** ^1^ULR 4354 - CIREL - Centre Interuniversitaire de Recherche en Education de Lille, Univ. Lille, Lille, France; ^2^Laboratoire Interdisciplinaire en Neurosciences, Physiologie et Psychologie, Université Paris Nanterre, Nanterre, France; ^3^Laboratoire VCR, Equipe d'accueil Religion, Culture et Société, École de Psychologues Praticiens de L'Institut Catholique de Paris, Paris, France; ^4^APEMAC UR 4360 Université de Lorraine, Metz, France; ^5^Institut de Recherche Biomédicale des Armées (IRBA), Brétigny, France; ^6^UNI-FlowLab, University of Northern Iowa, Cedar Falls, IA, United States; ^7^KU Leuven, Itec Research Group at Campus Kulak Kortrijk, Leuven, Belgium; ^8^Centrale Lille, University of Lille, Lille, France; ^9^Quality of Life Research Center, Claremont Graduate University, Claremont, CA, United States

**Keywords:** flow, optimal experience, autotelic experience, loss of self-consciousness, adult learning, adult education, motivation, well-being

In the original article, there was an error in the Supplementary Material as published. There were spelling mistakes in the English translations of some of the terms in the Supplementary Material (p. 2).

A correction has been made to:

FlowD1a Je me sens capable de faire face aux exigences élevées de la situation.

*[I trust my ablity to meet the high demands of the situation]*.

*Correction [I trust my ability to meet the high demands of the situation]*.

A correction has been made to:

FlowD1b Je sens que je contrôle parfaitement mes actions.

*[I feel completly in control of my actions]*.

*Correction [I feel completely in control of my actions]*.

The authors apologize for this error and state that this does not change the scientific conclusions of the article in any way. The original article has been updated.

