# Supplementary material for: Optimal Experience in Adult Learning: Conception and Validation of the Flow in Education Scale (EduFlow-2)
Source: Front Psychol. 2021 Dec 30;12:828027. doi: 10.3389/fpsyg.2021.828027 (PMC8773090; doi:10.3389/fpsyg.2021.828027)
Supplement: Supplementary file 1 [file Data_Sheet_1.pdf]

Échelle de flow en contexte éducatif v.2 (EduFlow-2)

Lisez attentivement chaque phrase et répondez, sur l'échelle située en face, en entourant un nombre correspondant le mieux à ce que vous pensez 1 = pas du tout d'accord à 7 = tout à fait d'accord.

pas du tout d'accord	très peu d'accord	un peu d'accord	Moyennement d'accord	Assez d'accord	Fortement d'accord	tout à fait d'accord
1	2	3	4	5	6	7

[strongly disagree] <---> [totally agree]

En général, *quand je suis dans une activité (formation en ligne, travail en classe ou devoirs à la maison...) à contextualiser en fonction de l'activité… [During a learning activity…]*

01 D1a	Je me sens capable de faire face aux exigences élevées de la situation.	1 2 3 4 5 6 7
02 D2a	Je suis totalement absorbé·e par ce que je fais.	1 2 3 4 5 6 7
03 D3a	Je ne suis pas préoccupé·e par ce que les autres pourraient penser de moi.	1 2 3 4 5 6 7
04 D4a	J'ai le sentiment de vivre un moment enthousiasmant.	1 2 3 4 5 6 7
05 D1b	Je sens que je contrôle parfaitement mes actions.	1 2 3 4 5 6 7
06 D2c	Je ne vois pas le temps passer.	1 2 3 4 5 6 7
07 D3b	Je ne suis pas préoccupé·e par le jugement des autres.	1 2 3 4 5 6 7
08 D4b	Cette activité me procure beaucoup de bien-être.	1 2 3 4 5 6 7
09 D1c	A chaque étape, je sais ce que je dois faire.	1 2 3 4 5 6 7
10 D2b	Je suis profondément concentré·e sur ce que je fais.	1 2 3 4 5 6 7
11 D3c	Je ne suis pas inquiet·e de ce que les autres peuvent penser de moi.	1 2 3 4 5 6 7
12 D4c	Quand j'évoque cette activité, je ressens une émotion que j'ai envie de partager.	1 2 3 4 5 6 7

- FlowD1 : Contrôle cognitif/Cognitive control
- FlowD2 : Immersion et altération de la perception du temps/Immersion and Time transformation
- FlowD3 : Absence de préoccupation à propos du soi/Loss of self-consciousness
- FlowD4 : Expérience autotélique (Bien-être procuré par l'activité)/Autotelic experience (well-being provided by the activity)

Note : FlowD1+FlowD2+FlowD3 = Absorption cognitive/Cognitive absorption

Merci d'utiliser cette référence pour citer l'échelle de flow en éducation v.2 (EduFlow-2)

Heutte, J., Fenouillet, F., Martin-Krumm, C., Gute, G., Raes, A. Gute, D., Bachelet, R. & Csikszentmihalyi, M. (2021). Optimal Experience in Adult Learning: Conception and Validation of the Flow in Education Scale (EduFlow-2) *Frontiers in Psychology, section Educational Psychology, 12*, 1-12. https://doi.org/10.3389/fpsyg.2021.828027

Echelle de flow en éducation v.2 *[Flow in Education scale v.2]* (EduFlow-2)

FlowD1a	Je me sens capable de faire face aux exigences élevées de la situation.<br>*[I trust my ability to meet the high demands of the situation.]*
FlowD1b	Je sens que je contrôle parfaitement mes actions.<br>*[I feel completely in control of my actions.]*
FlowD1c	A chaque étape, je sais ce que je dois faire.<br>*[At each step, I know exactly what I have to do.]*
FlowD2a	Je suis totalement absorbé·e par ce que je fais.<br>*[I am wholly absorbed in what I am doing.]*
FlowD2b	Je suis profondément concentré·e sur ce que je fais.<br>*[I am deeply focused on what I am doing.]*
FlowD2c	Je ne vois pas le temps passer.<br>*[I am losing track of time.]*
FlowD3a	Je ne suis pas préoccupé·e par ce que les autres pourraient penser de moi.<br>*[I don't care about what others may think of me.]*
FlowD3b	Je ne suis pas préoccupé·e par le jugement des autres.<br>*[I am not concerned about the judgement of others.]*
FlowD3c	Je ne suis pas inquiet·e de ce que les autres peuvent penser de moi.<br>*[I am not worried about what others might think of me.]*
FlowD4a	J'ai le sentiment de vivre un moment enthousiasmant.<br>*[I have the feeling I am living a very exciting experience.]*
FlowD4b	Cette activité me procure beaucoup de bien-être.<br>*[This activity brings me a sense of well-being.]*
FlowD4c	Quand j'évoque cette activité, je ressens une émotion que j'ai envie de partager.<br>*[When I talk about this activity, I feel such a deep emotion that I want to share it.]*

- FlowD1 : Contrôle cognitif/Cognitive control
- FlowD2 : Immersion et altération de la perception du temps/Immersion and Time transformation
- FlowD3 : Absence de préoccupation à propos du soi/Loss of self-consciousness
- FlowD4 : Expérience autotélique (Bien-être procuré par l'activité)/Autotelic experience (well-being provided by the activity)

Note : FlowD1+FlowD2+FlowD3 = Absorption cognitive/Cognitive absorption

Please use this reference to cite the Flow in Education scale v.2 (EduFlow-2)

Heutte, J., Fenouillet, F., Martin-Krumm, C., Gute, G., Raes, A. Gute, D., Bachelet, R. & Csikszentmihalyi, M. (2021). Optimal Experience in Adult Learning: Conception and Validation of the Flow in Education Scale (EduFlow-2) *Frontiers in Psychology, section Educational Psychology, 12*, 1-12. https://doi.org/10.3389/fpsyg.2021.828027
